# Recapitulating Zika Virus Infection in Vagina of Tree Shrew *(Tupaia belangeri)*

**DOI:** 10.3389/fcimb.2021.687338

**Published:** 2021-06-25

**Authors:** Zulqarnain Baloch, Zhili Shen, Li Zhang, Yue Feng, Daoqun Li, Na-Na Zhang, Yong-Qiang Deng, Chunguang Yang, Xiaomei Sun, Jiejie Dai, Zifeng Yang, Cheng-Feng Qin, Xueshan Xia

**Affiliations:** ^1^ Faculty of Life Science and Technology, Yunnan Provincial Center for Molecular Medicine, Kunming University of Science and Technology, Kunming, China; ^2^ State Key Laboratory of Pathogen and Biosecurity, Beijing Institute of Microbiology and Epidemiology, Beijing, China; ^3^ Key Laboratory of Animal Models and Human Disease Mechanisms, Kunming Institute of Zoology, Chinese Academy of Sciences, Center of Tree Shrew Germplasm Resources, Institute of Medical Biology, Chinese Academy of Medical Science and Peking Union Medical College, Kunming, China; ^4^ State Key Laboratory of Respiratory Disease, National Clinical Research Center for Respiratory Disease, First Affiliated Hospital of Guangzhou Medical University, Guangzhou, China

**Keywords:** vaginal infection, tree shrew, Zika, animal model, vaginal douching

## Abstract

Sexual transmission of Zika Virus (ZIKV) elevates the risk of its dissemination in the female reproductive tract and causes a serious threat to the fetus. However, the available animal models are not appropriate to investigate sexual transmission, dynamics of ZIKV infection, replication, and shedding. The use of tree shrew as a small animal model of ZIKV vaginal infection was assessed in this study. A total of 23 sexually mature female tree shrews were infected with ZIKV GZ01 *via* the intravaginal route. There was no significant difference in change of body weight, and the temperature between ZIKV infected and control animals. Viral RNA loads were detected in blood, saliva, urine, and vaginal douching. ZIKV RNA was readily detected in vaginal lavage of 22 animals (95.65%, 22/23) at 1 dpi, and viral load ranged from 104.46 to 107.35 copies/ml, and the peak of viral load appeared at 1 dpi. The expression of key inflammatory genes, such as IL6, 8, CCL5, TNF-a, and CXCL9, was increased in the spleen of ZIKV infected animals. In the current study, female tree shrews have been successfully infected with ZIKV through the vaginal route for the first time. Interestingly, at first, ZIKV replicates at the local site of infection and then spreads throughout the host body to develop a robust systemic infection and mounted a protective immune response. This small animal model is not only valuable for exploring ZIKV sexual transmission and may also help to explain the cause of debilitating manifestations of the fetus *in vivo*.

## Introduction

Zika virus, a member of the Flaviviridae family first reported in 1947 (Wikan and Smith, 2016), is an arthropod-based-vector-born virus (Epelboin et al., 2017). It is pathogenic to humans and nonhuman primates (Nugent et al., 2017), associated with self-limiting symptoms such as fever, muscle aches, rashes, conjunctivitis, arthralgia (Nugent et al., 2017; Almeida et al., 2020), unexpected clinical outcomes such as meningoencephalitis and Guillain-Barré syndrome in adults (Nugent et al., 2017), spontaneous abortion, microcephaly, and intrauterine growth restriction of the fetus (Nugent et al., 2017; Peregrine et al., 2019). Different ZIKV animal models such as rodents, sheep, pigs, hamsters (Zhang N. N. et al., 2016; Narasimhan et al., 2020), and non-human primates (NHPs) (Osuna et al., 2016; Mohr, 2018) have been developed. Previously reported ZIKV animal models have provided useful information to help us to understand the pathogenesis of infection, initial drug, and possible vaccine candidate screening. However, the anatomy, physiology, and brain size of these models are quite distinct as compared to humans particularly rodents. Therefore, rodent model is not always an appropriate model for ZIKV. Another leading unanticipated consequence of ZIKV infection is sexual transmission. Sexual transmission of ZIKV from female to male, male to female (Russell et al., 2017; Counotte et al., 2018), and from male to male partners have already been reported (Deckard et al., 2016). Sexual or intrauterine transmission of ZIKV is the main cause of debilitating manifestations such as abortion or microcephaly of fetus (Peregrine et al., 2019). Therefore, the application of rodent models to study the vertical or sexual transmission of ZIKV infection is further limited. NHPs are valuable models for investigation of the basic and applied research of human viral infections including ZIKV (Osuna et al., 2016; Mohr, 2018) but the application of NHPs in biomedical research is very limited (only one-half of the one percent) due to serious ethical and practical concerns (Friedman et al., 2017). Therefore, there is a critical need for alternative animal models of ZIKV that certainly develop infections like humans and can be a more appropriate animal model.

Tree shrew (*Tupaia belangeri*) is a small-sized mammal (Cao et al., 2003) and commonly populated in South, Southeast Asia, and the southern part of China (Schmitt et al., 2015). It is an extremely valuable animal model for studying human infectious diseases (Cao et al., 2003; Li et al., 2018). Tree shrew has unique characteristics such as low production cost, short reproductive and gestation period, and high brain-to-body mass ratio, etc. (Cao et al., 2003). Additionally, whole-genome analysis of Chinese tree shrew suggested that *Tupaia belangeri* are more closely related to humans and NHPs as compared to other animal models (Fan et al., 2013). Our group has already established tree shrew as ZIKV model by subcutaneous injection and described ZIKV’s infectivity in the primary cell-derived from different tissues of tree shrew (Zhang N. N. et al., 2019). Here for the first time, we have infected tree shrews with ZIKV through the vaginal route. Interestingly, adult female tree shrews developed a robust systemic infection and mounted a protective immune response although the symptoms of rashes are not distinct.

## Methods

### Ethics Statement

Chinese tree shrews (*Tupaia belangeri Chinensis*) (F1 generation) were acquired from the Kunming Institute of Zoology. In this study, all the animal related experiments were strictly accomplished following the instruction of Chinese Regulations of Laboratory Animals (Ministry of Science and Technology of the People’s Republic of China) and Laboratory Animal—Requirements of Environment and Housing Facilities (GB 14925-2010, National Laboratory Animal Standardization Technical Committee). Animal experiments were performed under sodium pentobarbital anesthesia. This study was approved by the Animal Experiment Committee of the Laboratory Animal Center, Faculty of Life Science and Technology, Kunming University of Science and Technology.

### Viruses and Cells

ZIKV strain GZ01 (GenBank accession number KU820898) was originally isolated from a Chinese male patient who returned from Venezuela (Zhang F. C. et al., 2016). ZIKV stocks were propagated in *Aedes albopictus* C6/36 cells and titrated by a plaque-forming assay in BHK-21, cells as previously described (Dai et al., 2016; Li X. F. et al., 2016). All experiments involving infectious ZIKV were conducted in biosafety level 2 (BSL2) facilities. All further details related to cell culture and virus purification has been previously described (Zhang N. N. et al., 2019).

### Animal Experiments

**Figure 1:**
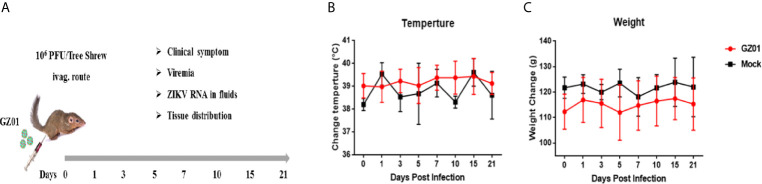
**(A)** Experimental design and sampling index. **(B)** Changes in body temperature. **(C)** Body weight of tree shrews after ZIKV infection.

A total of 23 sexually mature female tree shrews were infected with 10^5^ or 10^6^ PFU of ZIKV GZ01 *via* the intravaginal route (**Figure 1A**). Four animals were inoculated with phosphate Buffer Saline and Heat-inactivated ZIKV *via* vaginal route as controls. All the animals were regularly monitored for 21 days for body clinical symptoms such as fever, skin rashes, behavior, and weight change. Blood, saliva, douche, and urine samples were collected at 1, 2, 3, 5, 7, 10, 15 dpi for viral load analysis (Zhang N. N. et al., 2019). The remaining method has been previously described (Zhang N. N. et al., 2019). Vaginal douching was performed to collect vaginal secretion. All collected samples were stored at −80°C till further use. Animals were given pentobarbital intramuscularly before euthanasia. Detail of animal related experiments such as liver, spleen, lung, kidney, ovary, uterus, vagina, vulva, bladder, muscle, and skin samples collection, PCR, and histopathological analysis has been already explained in our pervious study (Zhang N. N. et al., 2019).

### RNA Extraction, RT-qPCR, and Cytokines Expression Analysis

Total RNA from blood, urine, saliva, douching, and organ samples were extracted using TRIZOL reagent (Life Technologies) according to the manufacturer’s recommendations. Total RNA concentration and quality was measured with agarose-gel electrophoresis and NanoDrop2000 spectrophotometer (NanoDrop; Thermo Fisher Scientific, Wilmington, DE, USA) respectively. The first-strand cDNA was produced with a High-Capacity cDNA Archive Kit (Applied Biosystems) by using 1 μg RNA as templet per reaction.

In this study, a probe and virus-specific primers were used that has been previously described (Koide et al., 2016; Li X. F. et al., 2016). A One Step PrimeScript™ RT-PCR Kit (064A, TaKaRa, Japan) was used to perform RT-qPCR on a LightCycler system (Roche, USA). The viral titer for organ weight and volume for reporting the organ loads as RNA copies/gram and RNA copies/ml were adjusted. While SYBR green RT-qPCR mix (TaKaRa, Japan) was used according to manufacturer’s recommendation to measure various cytokines mRNA levels (Zhang N. N. et al., 2019).

The expression of cytokines in the cell lysates was measured at a different time of post-infection by qRT-PCR with GAPDH as a housekeeping control gene. Primers used RT-qPCR reactions are available on request. Further experimental detail has been previously described (Zhang L. et al., 2019).

### Statistical Analysis

We used GraphPad Prism Software version 5.01 (GraphPad Software Inc., La Jolla, CA, USA) for statistical data analysis. A log-rank test was applied for Survival curves comparison. Unpaired and Student’s t-test was performed for statistical evaluation. P < 0.05 was considered to be statistically significant.

## Results

### ZIKV Vaginal Infection Causes No Clinical Symptoms in Tree Shrew

After vaginal infection, the body weight and temperature were regularly monitored. There was no change of body mass and body temperature (Tb) between ZIKV infected and control group (**Figure 1B**). No dermatological manifestations were observed in ZIKV-infected group as compared to the control throughout the study period (**Figure 1C**).

### Viral RNA Loads in Blood, Vaginal Douching, Urine, and Saliva After ZIKV Vaginal Infection in Tree Shrew

**Figure 2:**
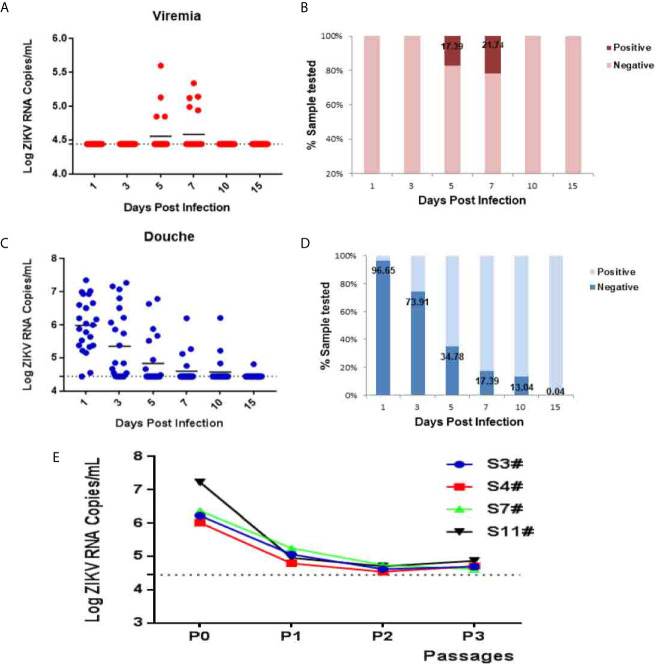
ZIKV establishes systemic infection in tree shrews infected *via* vaginal inoculation. Viral loads in blood and vaginal douching were determined by RT-qPCR. **(A, B)** The kinetics and time courses of viremia in blood of ZIKV-infected tree shrews. **(C, D)** The kinetics and time courses of the viral load in vaginal douching of infected tree shrews. The detection limit is indicated by the dotted line. **(E)** C6/36 cell were used to isolate the virus from the douche of ZIKV infected tree shrews 1 dpi. The symbol “#” means number.

To further characterize ZIKV vaginal infection dynamics in tree shrews, blood, saliva, urine, and vaginal douching samples were collected from animals and subjected to virological assays. A transient low-load viremia appeared at 5–7 dpi (**Figure 2A**), and the peak of viremia appeared at 5 dpi (**Figure 2A**). ZIKV viremia was detected in 17.39% (4/23) tree shrews at 5 dpi and 21.74% (5/23) at 7dpi, and the viral loads in serum ranged from 10^4.85^ to 10^5.6^ copies/ml (**Figure 2B**) and became undetectable at 10 dpi.

Following vaginal infection, ZIKV viremia was readily detected in vaginal lavage of 22 animals 95.65% (22/23) at 1 dpi and viral load ranged from 104.46 to 10^7.35^ copies/ml. The peak of viremia appeared at 1 dpi (**Figure 2C**). Interestingly, a transient high-load viremia (10^4.46^ to 10^6.10^ copies/ml) persisted at 10 dpi in three animals 13.04% (3/23) except that of one animal that still exhibited delayed viremia having a low level at 15 dpi (**Figure 2D**). Furthermore, to confirm the production of the infectious progeny of ZIKV virions, vaginal lavage at 1 dpi was collected and subjected to virus isolation in mosquito C6/36 cells (**Figure 2E**).

**Figure 3:**
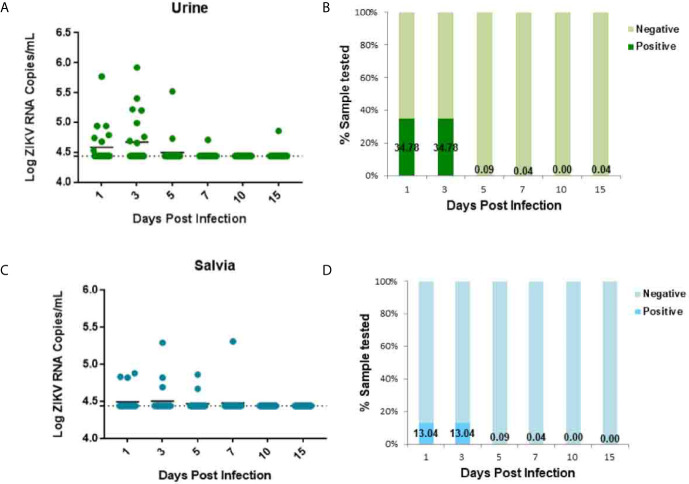
ZIKV establishes systemic infection in tree shrews infected *via* Vaginal inoculation. Viral loads in urine and saliva were determined by RT-qPCR. **(A, B)** The kinetics and time courses of viremia in urine of ZIKV-infected tree shrews. **(C, D)** The kinetics and time courses of the viral load in saliva in infected tree shrews. The detection limit is indicated by the dotted line.

Urine and saliva samples were collected from animals for further characterization of ZIKV infection dynamics. Following vaginal inoculation, a transient low-load viremia appeared at 1–3 dpi in the urine of ZIKV infection (**Figure 3A**). The peak of viremia appeared at 3 dpi (**Figure 3A**). One animal also exhibited delayed viremia having a low level at 15 dpi (**Figure 3A**). ZIKV secretion in urine detected in 34.78% (8/23) tree shrews at 1 dpi and 34.78% (8/23) at 3 dpi **Figure 3B**, and the viral loads in urine ranged from 10^4.40^ to 10^5.92^ copies/ml, and the lavage fluid was 10^4.46^–10^7.35^ copies/ml, the viral load of urine is an order of magnitude lower than that of lavage fluid, but the time of infection extended to 15 dpi. ZIKV secretion was radially detected at 1–7 dpi (**Figure 3C**) but the detection situation in the saliva is irregular. ZIKV secretion in saliva was detected in 13.04% (3/23) tree shrews at 1 dpi and 13.04% (3/23) at 3 dpi (**Figure 3D**).

### Tissue Distribution After Vaginal Infection of Tree Shrew With GZ01 Strain

**Figure 4:**
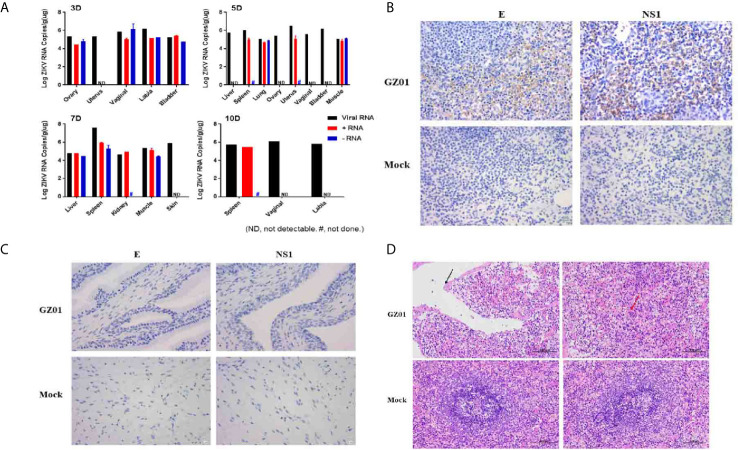
**(A)** Viral loads in different tissues of ZIKV infected tree shrews were detected by RT-qPCR. **(B)** IHC detected ZIKV proteins of the spleen of tree shrew 7dpi, Positive viral proteins were shown in brown and DAPI in blue. **(C)** Histopathological changes in the spleen of tree shrew 7 dpi. The cavities are surrounded by thin connective tissue (black arrows), and macrophages proliferate in the red pulp (red arrows). **(D)** IHC detected ZIKV proteins of the vagina of tree shrew 3 dpi, Positive viral proteins were shown in brown and DAPI in blue.

To determine the ZIKV replication and tissue tropism in tree shrews infected with ZIKV, liver, spleen, lung, kidney, ovary, uterus, vagina, vulva, bladder, muscle, and skin samples were collected. In this study, viral RNA was found in the liver, spleen, lung, kidney, ovary, uterus, vagina, vulva, bladder, muscle, and skin (**Figure 4A**) which indicates that ZIKV vaginal infection has been established as a systemic multi-tissue and multi-organ infections. In brief, viral RNA was only detected in reproductive organs or tissues (Ovary, Uterus, Vaginal, Labia, Bladder) at 3 dpi (**Figure 4A**) and viral RNA was detected in liver, spleen, lung, ovary, uterus, vagina, bladder, and muscle at 5 dpi. While, viral RNA was only found in the liver, spleen, kidney, muscle, and skin at 7 dpi and viral RNA was found only in the spleen, vagina, and labia at 10 dpi (**Figure 4A**). ZIKV RNA was persistently detected in the spleen at 5–10 dpi, with the highest viral load of 10^7.63^ copies/g at 7 dpi.

ZIKV is a single-stranded positive-strand RNA virus and the negative-strand RNA is the intermediate of viral nucleic acid replication. Therefore, the detection of negative-strand RNA is an important indicator of replication of ZIKV. In this study, both positive and negative-RNA strand of ZIKV was detected in different tissues and organs, with high RNA concentration. Negative-strand RNA virus was detected in the ovaries, vagina, vulva, and bladder at 3 dpi, in lungs and muscles at 5 dpi, and in the liver, spleen, and muscle at 7 dpi. The viral load of viral RNA, positive-strand RNA, and negative-strand RNA in the spleen can reach 10^7.63^, 10^5.99^, and 10^5.55^ copies/g, respectively (**Figure 4A**).

As in this study, a high viral load of ZIKV was detected in the spleen and vaginal tissues at 7 dpi after the successful vaginal infection. Then, immunostaining of spleen and vaginal tissues were performed with convalescence serum from a recovered ZIKV patient and the mouse-derived NS1 protein produced in our laboratory. ZIKV antigens were predominantly detected in the spleen (**Figure 4B**) and not in vaginal tissues (**Figure 4C**). Further, it was found that the spleen tissue was atrophic, the capsule was thickened, uneven, and the number of spleen nodules was reduced. Irregular cavities were seen in the middle of the spleen, and these cavities were wrapped by thinner connective tissues and macrophages with increased the red pulp (**Figure 4D**).

### Detection of Tissue Cytokines After Vaginal Infection of GZ01 Virus in Tree Shrew

**Figure 5:**
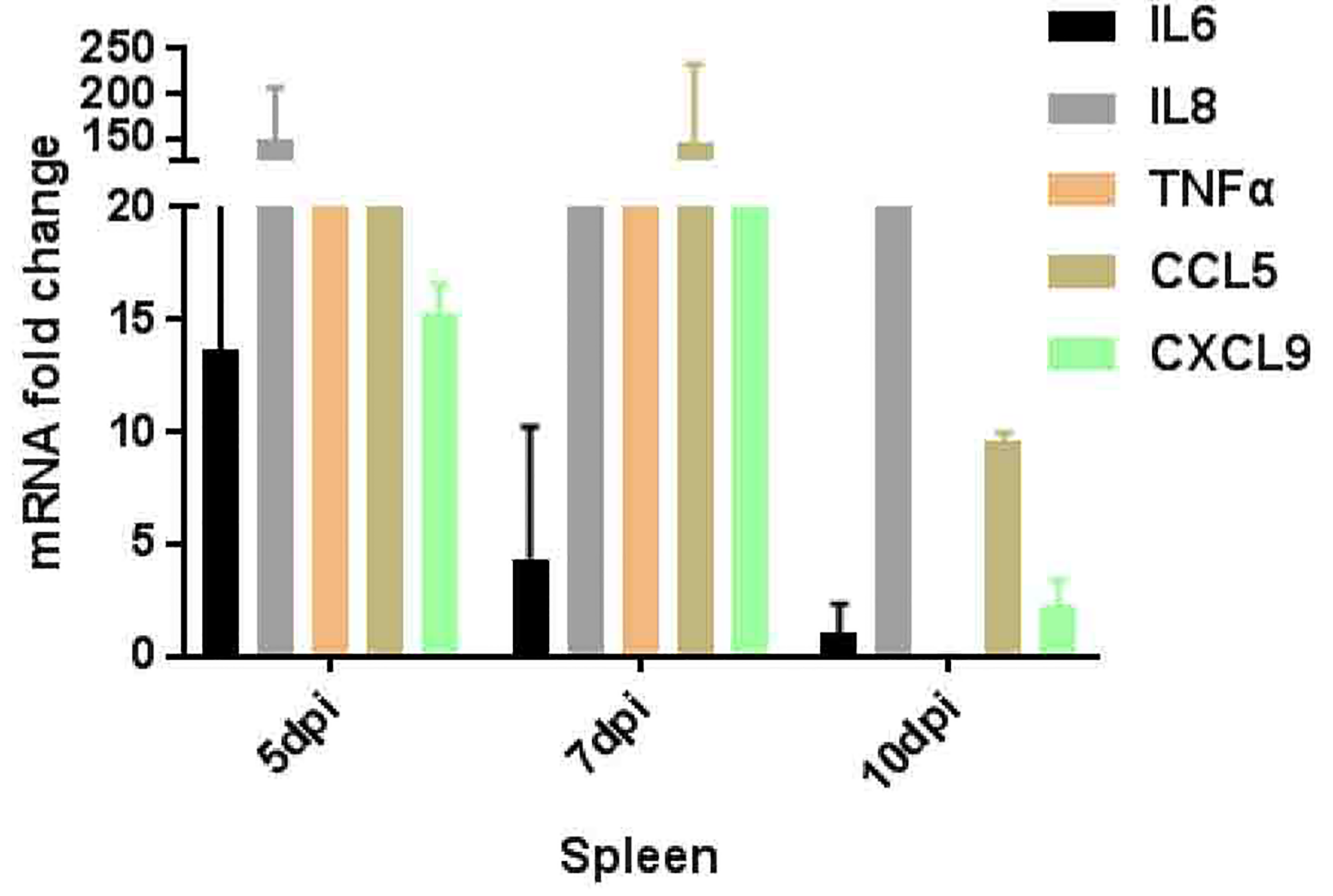
Inflammatory genes expression pattern in spleen of tree shrews infected ZIKV *via* vaginal route.

Different types of immune cells secrete a variety of cytokines after viral infection. Therefore, a variety of cytokines such as IL-6, IL-8, TNF-α, IL-8 (CXCL8), and CXCL9 were further detected (**Figure 5**) which participate in or mediate the antiviral immune response, inflammatory response, and primer sequences are available on request. In this study, the expression of IL-6 peaked at 5 dpi and decreased as the virus was gradually cleared out of the body. IL-8 (CXCL8) was significantly up-regulated at 5–10 dpi, and the expression level of CXCL9 showed a trend of first increasing and then decreasing at 5–10 dpi, which was consistent with the trend of viral load in the spleen. While, TNF-α which is a marker of viral infection, stimulates T-lymphocytes to produce a variety of inflammatory factors, thereby promoting the occurrence of inflammatory reactions, and their expression also tends to increase at 5–7 dpi.

## Discussion

There are different animal models available to study the pathogenesis of ZIKV infection (Zhang N. N. et al., 2016; Abdullah et al., 2019). However, application of these models is limited due to their genetic variation, high cost of production, ethical concern, specific facilities, and workers needed (Montagutelli and Barré-Sinoussi, 2015; Julander, 2016). A Tree shrew model of viral infections shows a potential to replace other models due to genetic and physiologic similarities among NHPs, human, and tree shrews (Cao et al., 2003; Li et al., 2018; Abdullah et al., 2019). Additionally, tree shrews have already been successfully developed as animal models of different viral infections including influenza (Yang et al., 2013), herpes simplex (Li L. et al., 2016), hepatitis C virus (Feng et al., 2017), and ZIKV (Abdullah et al., 2019). Successful establishment of the tree shrew model of ZIKV has been demonstrated by SC inoculation (Shuaib et al., 2016; Abdullah et al., 2019). In this study, all animals were regularly monitored for Tb, animal weight, etc. throughout the experiment period. There was no significant change in animal Tb, weight, and animal behavior. The observations of this study are consistent with previously reported observations (Zhang N. N. et al., 2019). Additionally, there were no skin rashes, deaths, and the body coat was also normal. All animals were agile. Results showed that ZIKV vaginal infection did not affect the normal life of tree shrews.

Viremia is a typical marker of flavivirus replication in the host body (Li X. F. et al., 2016). To establish tree shrew as a ZIKV vaginal infection model, 23 tree shrews were challenged *via* a vaginal route, and the samples were collected from blood, urine, saliva, and vaginal douching at days 1, 3, 5, 7, 10, 15. A low-load transient viremia was detected with RT-qPCR in blood at 5–7 dpi (10^5.6^ RNA copies/ml), which peaked at 7 dpi and disappeared. In Rhesus monkeys after vaginal inoculation, viremia was detected at 4–6 dpi, which peaked at 6–10 dpi (Li X. F. et al., 2016). A transient viremia (10^6.15^ RNA copies/ml) was detected at 1 dpi in immunocompetent mice after SC inoculation (Zhang N. N. et al., 2016). Similarly, a constant viremia (10^4.4^–10^6.02^ RNA copies/ml) was detected from 2 to 5 dpi in Guinea pigs infected *via* a subcutaneous and vaginal route which peaked at 3 dpi (Saver et al., 2019). Current study viremia initiation is consistent with detection of ZIKV viremia in Rhesus monkeys (Li X. F. et al., 2016), mice (Zhang N. N. et al., 2016), and Guinea pigs (Saver et al., 2019) but the peak of viremia is only comparable with Rhesus monkeys (Li X. F. et al., 2016). ZIKV shedding in urine, salvia, a low load transient viremia was detected at 1–7 dpi and peaked at 3 dpi which is consistent with the detection of viremia in mice (Zhang N. N. et al., 2016) and tree shrews (Zhang N. N. et al., 2019).

The dynamics of ZIKV shedding in vaginal douching was assessed. High-level viremia was detected at day 1 followed by days 3, 5, 7, 10, 15, and a peak of viremia was achieved at day 1 ranged from 10^4.46^ to 10^7.35^ copies/ml. ZIKV RNA remained detectable on day 15. Further, the infectious ZIKV from vaginal douching was recovered from 1 to 3 dpi. C6/36 cells were infected to isolate infectious ZIKV. After 7 days of culture, the supernatant was taken for quantitative detection. Comparing the virus copy number of different generations with the virus copy number of the initial lavage fluid, a sample titer 103 times high as compared to the copy number of original virus was selected for virus isolation. This showed that there is an infectious live virus in the vaginal douching fluid which produces effective replication on C6/36 cells. Unfortunately, the infectious live virus has not been successfully isolated from the 1 dpi vaginal douching fluid. The reason may be that the most of the virus in the vagina at day 1 was the residual virus.

Ovary, uterus, vagina, vulva, bladder, muscle, liver, spleen, lung, kidney, and skin are the possible site of virus replication. Further, ZIKV RNA copy numbers, +ve and –ve strand were detected in different body tissues to verify virus replication. Mainly, ZIKV RNA load +ve and –ve strand were detected in reproductive organ at 3–5 dpi but spleen was the organ with ZIKV RNA load, +ve and –ve strand numbers at 5–10 dpi. Observations of this study are consistent with the Guinea pigs model (Saver et al., 2019), as high viral load was detected in the spleen and vaginal tissues. Therefore, immunostaining of spleen and vaginal tissues were performed. Histopathology of the spleen and vaginal tissues showed that lesions generally appeared to be distributed more diffusely throughout the spleen but not in vaginal tissues.

Inflammation is an important marker of the natural immune action against infection (Ali et al., 2020). Key inflammatory genes, such as IL6, 8, CCL5, TNF-a, and CXCL9 by RT-qPCR expression were further validated. Results showed a strong antiviral immune resistance against ZIKV infection. The expression of IL6, TNF-a, CCL5, and CXCL9 in the spleen was up-regulated, especially the expression of CCL5 and CXCL9 showed a trend of first increasing and then decreasing at 5–10 dpi, in line with the trend of viral load in the spleen (Zhang N. N. et al., 2019). Further, TNF-αexpression is high which may induce endothelial barrier dysfunction (Dewi et al., 2004; Zhang L. et al., 2019). These results showed active and recruit immune cells movement toward the sites of infection (Avirutnan et al., 1998; Zhang L. et al., 2019).

## Conclusion

In this study, the female tree shrew as ZIKV vaginal infection model has been successfully established. Interestingly, at first, ZIKV replicates at the local site of infection, and then spreads throughout the host body to develop a robust systemic infection and mounted a protective immune response. This small animal model is not only valuable for exploring ZIKV sexual transmission, but may also help to explain the cause of debilitating manifestations of the fetus *in vivo* and also a useful platform to characterize the novel therapeutics against ZIKV.

## Data Availability Statement

The original contributions presented in the study are included in the article/supplementary material. Further inquiries can be directed to the corresponding authors.

## Ethics Statement

In this study, all the animal related experiments were strictly accomplished following the instruction of Chinese Regulations of Laboratory Animals (Ministry of Science and Technology of the People’s Republic of China) and Laboratory Animal—Requirements of Environment and Housing Facilities (GB 14925-2010, National Laboratory Animal Standardization Technical Committee). Animal experiments were performed under sodium pentobarbital anesthesia. This study was approved by the Animal Experiment Committee of the Laboratory Animal Center, Faculty of Life Science and Technology, Kunming University of Science and Technology.

## Author Contributions

All authors contributed to the concept of this study. XX and C-FQ designed the study. ZB, ZS, LZ, YF, DL, N-NZ, Y-QD, CY, XS, JD, and ZY performed study, acquired and analyzed data. XX and ZB wrote the manuscript. All authors contributed to the article and approved the submitted version.

## Funding

The work was supported by grant from National Natural Science Foundation of China (No. U1702282).

## Conflict of Interest

The authors declare that the research was conducted in the absence of any commercial or financial relationships that could be construed as a potential conflict of interest.
